# Understanding the Determinants for the Adoption of Mobile Market Research: An Empirical Study in the Spanish Market Research Industry

**DOI:** 10.3389/fpsyg.2020.00288

**Published:** 2020-03-06

**Authors:** Carmen Pacheco-Bernal, Ana Isabel Jiménez-Zarco, María-Jesús Martínez-Argüelles

**Affiliations:** Faculty of Economics and Business, Universitat Oberta de Catalunya (UOC), Barcelona, Spain

**Keywords:** mobile market research, technology adoption, TOE framework, PLS analysis, market research industry, Spain

## Abstract

The aim of this study is to investigate the determinants for the intention to adopt mobile technology as a data collection methodology in market research projects. A conceptual framework was developed using the technology-organization-environment (TOE) model to identify technological factors (perceived benefits and limitations), organizational factors (open attitude toward change, professional competence, satisfaction with traditional systems, and firm size), and environmental factors (industry pressure, client pressure, and participant pressure) affecting adoption. The empirical study was performed with data from 67 firms in the Spanish market research industry, which were analyzed using partial least squares (PLS). The results suggest that only organizational and environmental factors have a significant influence on adoption. Key factors include professional competence, organizational openness, satisfaction with traditional and online methodologies, and pressure from industry, clients and survey participants. The findings reveal that technological characteristics are no longer a driver, as firms are starting to adopt mobile marketing research based on its greater convenience for participants, and as an element of strategic differentiation.

## Introduction

Just as the mobile revolution has had a profound impact on society, creating new, and modifying existing economic activities, so too has the market research industry undergone a similar transformation (Shankar et al., 2010; Grewal and Levy, 2016). Mobile market research is now one of the technological innovations having the greatest impact on firms’ marketing information systems. The opportunities offered by mobile technology as a data collection method have been pointed out both in academic research (Callegaro, 2013; Antoun, 2015; Couper et al., 2017) and in the market research industry itself (Macer and Wilson, 2011; ESOMAR European Society for Opinion and Marketing Research, 2015; GreenBook, 2015), identifying it as a new and important technological innovation in data collection.

There are numerous advantages to using mobile techniques for market research purposes. As well as the usual benefits of online marketing research, such as lower costs and increased speed in obtaining data (Malhotra and Peterson, 2001; Schonlau et al., 2001; Ilieva et al., 2002), mobile technology also offers greater convenience for participants, captures geolocation data, and facilitates more personal ways of reaching participants (Macer and Wilson, 2009). Furthermore, mobile surveys can be used for in-the-moment data collection, barcode scanning and visual data capture (pictures and videos). Given these benefits, academics and professionals have both agreed that mobile surveys would likely become the most widely used data collection method (Cameron and Weisberg, 2003; Friedrich-Freksa and Liebelt, 2005; Robbins, 2011).

Yet despite the benefits of mobile methods to the research industry, compared to more traditional techniques, their adoption and diffusion as a data collection method are still fairly limited. In 2017, mobile research represented 9% of the total spend on research methods (ESOMAR European Society for Opinion and Marketing Research, 2018), while it was chosen as the main information collection method in only 5% of projects (GreenBook, 2018). These data highlight how mobile methodologies have failed to become the most widely used technique in the industry, contrary to the predictions of academics and professionals. Through technological innovations’ adoption and diffusion framework, our research seeks to understand the process of adopting mobile methodologies and identify factors that affect (by either facilitating or inhibiting) the industry’s decision to adopt mobile marketing research in Spain.

## Literature Review

### Mobile Market Research

Mobile market research refers to a set of online methods or techniques using electronic mobile devices connected to the Internet to gather participants’ answers in market research surveys (Poynter et al., 2014). Until relatively recently, mobile market research included only research conducted via, or about mobile phones. However, since the arrival of new mobile devices (such as tablets and phablets), the term has broadened to cover the following methods: (1) Online quantitative research, whereby participants complete surveys on their mobile devices or download an app on their devices, which collects information on their environment, i.e., passive data collection in which mobile devices gather data without participants’ active intervention; (2) Online qualitative research, involving participation via mobile devices (e.g., taking part in an online focus group or in an online marketing research community), or in which data are collected from participants (e.g., photos and recordings of participants’ experiences with the products and services being tested), as in ethnographic studies; 3. Some authors (such as Poynter et al., 2014) also include face-to-face surveys, in which answers are collected via mobile devices (mobile computer-aided personal interviewing, mCAPI) and telephone surveys, which interviewees answer using their mobile phones (mobile computer-aided telephone interviewing, mCATI).

Mobile devices used in market research are varied and constantly evolving, their most important feature being the collection of answers (via questionnaires) or data (via techniques for observing or monitoring users) from the research subjects. Today, mobile phones and tablets are the most commonly used devices (Callegaro et al., 2015; Wells, 2015), though phablets and wearable technology are also growing in importance (GreenBook, 2018).

In the early years of mobile device use as a mode for collecting information from participants, both academic researchers and professionals agreed that the innovation would offer highly attractive opportunities for social research (Teo and Pok, 2003; Callegaro et al., 2004; Vehovar et al., 2004). Likewise, new uses and applications for mobile devices to collect market research data have been developed in recent years. Evidence suggests such ‘in-the-moment’ research (at the precise moment of purchase in a store, eating in a restaurant or staying at a hotel) provides deeper, more precise and more accurate information on consumers than that obtained after the purchase or consumption act (Poynter et al., 2014; Wells et al., 2014; Wells, 2015), as information is still fresh in people’s minds. In addition, market research using passive data collection systems on mobile devices enables large amounts of information to be collected while participants go about their daily activity, without requiring their active participation. Mobile devices have also helped in the development of new approaches in ethnographic research (Vargo and Lusch, 2004). Researchers can assign tasks to interviewees, whereby they record their thoughts and actions by taking photos during the behavior under research, or by making audio and video recordings on their devices, which are then analyzed in order to understand their motivations and behaviors (Prahalad and Ramaswamy, 2004; Evans and Wolf, 2005; Cook, 2008).

During the last decade, academic literature on mobile market research has focused mainly on the methodological aspects of the technique. Maxl et al. (2009) concur in stating that the development and application of mobile methodologies seem to be driven mainly by industry researchers, not academics (Mobile Marketing Research, p. 9). Indeed, initially, academic literature focused on analyzing the advantages and limitations of mobile techniques for obtaining information (Macer and Wilson, 2009), while it now focuses on ways of optimizing the design of questionnaires distributed on mobile devices (Li and Townsend, 2008; Callegaro, 2010). Subsequently, researchers turned their attention to designing experiments to analyze the differences between answers to surveys provided via mobile devices and those provided via PC, laptops and tablets (Buskirk and Andres, 2012; Mavletova and Couper, 2013). More recently, the literature has turned to topics such as new environments or platforms for quantitative mobile research, such as market research communities (Poynter et al., 2014; Mavletova and Couper, 2015), enriching information provided by participants using geolocation services, collaborating in ethnographic studies, and applying emerging mobile technologies, such as wearable technology, geo-fencing and virtual reality (Macer and Wilson, 2017).

However, there has been little interest from academia in the analysis of determinants, which explain the limited adoption and diffusion of mobile market research. Theories on the adoption and diffusion of technological innovations provide an appropriate, though yet unexplored, theoretical framework to identify and examine variables that determine business decisions on the adoption and use of mobile market research.

### The TOE Framework as a Technological Innovation Adoption Model

A variety of behavioral models and theories have been developed in order to understand and explain factors influencing the adoption and diffusion of technology. Indeed, analysis of new technology adoption has been one of the main lines of research in the information systems literature (Rogers, 1983; Kwon and Zmud, 1987; Swanson, 1988). Models for the adoption of technological innovations have helped create a theoretical framework that aids the adequate use of these innovations. They mainly analyze perceptions (among individuals and organizations) of determining factors in the use of technology and causal relationships between these factors and intention or adoption.

The most relevant theories and models for explaining the intention to use information systems, from the academic literature, are the innovation diffusion theory (IDT) (Rogers, 1983) and the technology acceptance model (TAM) developed by Davis (1989). However, several authors have shown the limitations of Rogers’ and Davis’ theoretical models, as they only consider the technological characteristics of the innovation (Molla and Licker, 2005; Rodríguez-Ardura and Meseguer-Artola, 2010; Ederington and McCalman, 2013; among others). A line of research stemming from this critique proposes the construction of new, more integrated models, thereby throwing light upon innovation adoption in businesses. This line is based on the work of Tornatzky and Fleischer who, in a study published in 1990, suggested an alternative, holistic approach to explaining innovation adoption in organizations. Their model, known as TOE (technology-organization-environment), consists of three contexts with a possible influence on the process of adopting or implementing technological innovation: the technological context, the organizational context, and the environmental context.

The first of these contexts is considered in Rogers’ IDT, which includes the technological attributes of the innovation and how firms perceive them. It focuses on how the characteristics of the innovation, associated with the technology itself, can influence its adoption, implementation and use. This assumes that only firms who perceive a relative advantage or potential benefit in the innovation will adopt it. Secondly, the organizational context consists of the characteristics of the firm (size, degree of centralization and technological readiness, management support, available resources, level of internationalization), assuming that all elements relative to organizational structure and processes can potentially facilitate or impede the adoption or effective implementation of an innovation in the firm. Thirdly, the environmental context refers to the environment in which the firm does business, considering the influence of the industry, competitive pressure, market regulations and relations with government agencies, among other factors.

In considering these three dimensions, the TOE model provides a useful theoretical framework for analyzing the technological innovation adoption and diffusion process in organizations. It is strengthened by solid theoretical foundations, robust empirical support and potential for application to all technological domains, although specific indicators in the three contexts (technology, organization and environment) vary from one study to another, depending on the contexts and subjects of analysis. The integrated theoretical framework developed by Tornatzky and Fleischer has been empirically tested in numerous studies on the ways in which firms adopt technological innovations, such as the Internet (Teo et al., 1997), open systems (Chau and Tam, 1997), e-commerce (Gibbs and Kraemer, 2004; Rodríguez-Ardura and Meseguer-Artola, 2010), e-business (Zhu et al., 2003, 2004; Oliveira and Martins, 2010), mobile customer relations management (mCRM) (San-Martin et al., 2016), software as a service (SaaS) (Martins et al., 2016; Oliveira et al., 2019), cloud computing (Low et al., 2011; Hsu et al., 2014), and a broad spectrum of general IS applications (Thong, 1999). The TOE model has been used to explain the adoption of innovations in a host of industries, including manufacturing (Zhu et al., 2006), retail, wholesale, technological and financial services (Zhu et al., 2004; Low et al., 2011). Furthermore, the TOE model has been tested in European, American, and Asian contexts (Gibbs and Kraemer, 2004; Zhu et al., 2006; Oliveira and Martins, 2010). The TOE theory permits analysis of the technological innovation adoption process by including different variables in the technological, organizational and environmental dimensions. In the technological context, the factors that most strongly determine the degree of technology adoption are perceived relative advantage/benefits and technological readiness. In the organizational context, the key factors are organizational competence, financial resources, and management support/innovative nature, while in the environmental context, the main predictive factor for adoption is competitive pressure.

## Research Model and Hypothesis

An integrated model of mobile market research adoption specifically for the market research industry was developed from the technological innovation literature. Each variable is discussed below.

### Mobile Market Research Intention to Adopt

In the behavioral literature, ‘use of the innovation’ and ‘intention to use the innovation’ are the two most common model-dependent variables (Turner et al., 2010; Wu and Du, 2012). Thus, some studies consider real use of technologies (Davis, 1989), while others consider intention to use or adoption as determining real use (Mathieson, 1991). Other authors include both concepts and suggest a causal relation between them (Davis et al., 1989; Taylor and Todd, 1995a, b). Following Fishbein and Azjen (1975) and Azjen (1991), in our research, the intention to adopt mobile market research is defined as the probability or predisposition of organizations to adopt or use mobile methods for data collection. The intention to use mobile market research will determine its acceptance and adoption, hence real use of mobile technology is not included in the research model, as intention to use has been shown to be a direct predictor for acceptance and use (Davis et al., 1989; Venkatesh et al., 2003), and therefore also applicable to the context of our study. Table 1, shows the construct used to measure the endogenous variable used in our model, “Intention to adopt mobile market research,” and its measurement items.

**TABLE 1:** Constructs and their measurement items.

**Perceived benefits**		
PB1	Answers are obtained quicker than with other techniques	Graham and Conry, 2011; Cunningham et al., 2013; Antoun, 2015; White and Stevens, 2015
PB2	Participants can answer anywhere and at any time	Macer and Wilson, 2009; Poynter et al., 2014
PB3	It reduces memory bias, as participants can answer while involved in the purchase or consumption experience (e.g., with in-the-moment questionnaires)	Macer and Wilson, 2009; Macer, 2012; Wells, 2015
PB4	Populations that are difficult to access by traditional methods can be reached (e.g., millennials, consumers in emerging countries)	Robbins, 2011; Antoun and Couper, 2013; SSI Survey Sampling International, 2015
PB5	It permits passive data collection	Poynter et al., 2014; Wells, 2015
PB6	New, richer approaches to consumer behavior are possible through ethnographic studies (usage diaries, video recordings, images, etc.)	Poynter et al., 2014
**Perceived limitations**		
PL1	Risk of breakoff is greater	De Bruijne and Wijnant, 2013; Mavletova, 2013
PL2	Questionnaires should be very short	Van Heerden et al., 2014; Turbina, 2014; Mavletova and Couper, 2015
PL3	Questionnaires have very simple designs	Macer and Wilson, 2009; Robbins, 2011; Van Heerden et al., 2014; Turbina, 2014; Mavletova and Couper, 2015
PL4	The stimuli are not well displayed on mobile screens	Poynter et al., 2014; Wells, 2015
**Professional competence**		
PC1	Our research team includes experts in mobile market research	Thong, 1999
PC2	Our researchers have a lot of experience in studies using mobile devices	
PC3	In the industry, we are leaders in mobile market research	
PC4	We have a high level of knowledge on how to conduct market surveys on mobile devices	Gangwar et al., 2015; and also Zhu and Kraemer, 2005; Tan et al., 2007; Lin and Lin, 2008
**Organizational openness**		
OO1	The organization is always looking for new ways of providing solutions	Siegel and Kaemmerer, 1978
OO2	Support for developing new ideas is readily available	
OO3	The organization is open to changes and adapts to them	
OO4	The management team is always looking for new, fresh ways to deal with problems	
**Satisfaction with traditional and online methodologies**		
SAT1	We are very satisfied with the broad coverage and representation provided by traditional techniques (face-to-face and telephone interviews)	Chau and Tam, 1997
SAT2	We are very satisfied with information collected via online questionnaires answered on desktops/laptops	
SAT3	The industry is still not clear about whether the insights provided by mobile research are better than those obtained from other methods	Item included in qualitative research
**Firm size**		
DIM1	Number of employees (Fewer than 10/10 or more employees)	Premkumar and Roberts, 1999; Thong, 1999; Zhu et al., 2003, 2004; Chang et al., 2007; Pan and Jang, 2008; Low et al., 2011; Chong and Chan, 2012; Junior et al., 2019
DIM2	Belonging to an international group (Yes/No)	Developments especially for research
DIM3	Having a proprietary and/or external consumer panel (Yes/No)	
**Pressure from industry**		
IP1	We have occasionally felt a degree of pressure from the industry to use mobile research in studies	Trainor et al., 2011
IP2	Most of our competitors are already offering mobile research-based studies	Wu et al., 2003; Wu and Lee, 2005
IP3	In the industry, anyone who does not offer mobile research-based methods falls behind	
IP4	In the industry, most firms will eventually adopt mobile research	
IP5	Disseminating good practices would probably help increase use of mobile research	Item included in qualitative research
IP6	In the industry there is still much to learn about mobile research	Item included in qualitative research
**Pressure from clients**		
CP1	Our clients are not asking us to use mobile research techniques	Srinivasan et al., 2002; Wu et al., 2003
CP2	We could lose clients if we did not use mobile research techniques in our studies	
CP3	We decided to use mobile research techniques because our clients expected it	
CP4	We believe that market survey providers should educate clients with regard to new research methods	Item included in qualitative research
**Pressure from participants**		
PP1	Increasingly people are using mobile devices to answer surveys	Item included in qualitative research
PP2	The fact that participants complete the survey on their mobiles, even though the questionnaire is not adapted to them, forces us to design responsive questionnaires	Ochoa and Castro, 2015
PP3	The fact that consumers always have their mobile on them helps the industry in using mobile research methods	Poynter et al., 2014
PP4	There is more engagement if interviewees can participate in the research using their mobile devices	
**Intention to adopt mobile market research**			
**Construct**	**Item**	**Measurement items**	**Source**

Intention to adopt mobile market research	ADO1	We intend to use mobile market research in the coming months	Davis et al., 1989; Venkatesh et al., 2003
	ADO2	We believe we will use mobile market research in our projects	

### Technological Context

Technological factors include the influence of the characteristics of the innovation, as has been frequently discussed in the literature on innovation adoption. One element widely considered to facilitate adoption is relative advantage, i.e., the degree to which an innovation is perceived as an improvement thanks to its benefits over the system it replaces (Rogers, 1983). Indeed, there is considerable evidence showing how adoption is positively influenced by the perceived benefits or relative advantages of an innovation with regards to alternative products or processes (Agarwal and Prasad, 1997; Srinivasan et al., 2002; Ramamurthy et al., 2008). Similarly, numerous studies based on an integrated perspective also show the influence of perceived benefits on the adoption of mobile CRM systems (San-Martin et al., 2016) and cloud computing (Low et al., 2011; Gangwar et al., 2015).

There are a variety of advantages associated with mobile market research when compared to other data collection methods, the first of which are the general benefits of online market research, such as lower costs and increased speed of data collection (Schonlau et al., 2001; Ilieva et al., 2002). Indeed, empirical evidence suggests that, in general terms, mobile surveys are usually answered within minutes of their reception (Graham and Conry, 2011; Cunningham et al., 2013). The ubiquity of mobile technology makes it easier for participants to answer anywhere and at any time (Macer and Wilson, 2009; Poynter et al., 2014). At the same time, it means that answers are closer in time and place, thereby reducing factors such as memory bias, as surveys are conducted at the same moment as the purchase or consumption act (Macer and Wilson, 2009; Macer, 2012). There is also a perception among industry experts that the answers are more genuine and accurate, given the highly personal nature of mobile devices (Alonso, 2013). A further benefit is that it enables additional information on the consumer’s experience, to be collected passively using automated monitoring and geolocation systems (Poynter et al., 2014; Wells, 2015). It also allows new kinds of consumer data to be collected, such as health data from wearable devices (Drew and Berney, 2015). A further advantage is that mobile research techniques facilitate new and richer approaches to consumer behavior in ethnographic studies, such as user diaries, and capturing visual data such as photos and videos (Poynter et al., 2014). As some authors have noted, participants become research partners, thereby producing greater engagement (Poynter et al., 2014; Wells, 2015). Furthermore, mobile technology helps reach target populations in places where traditional methods and other forms of online surveys would struggle to reach (Antoun and Couper, 2013). This is the case, for example, in developing countries, where use of non-mobile Internet access is less widespread and mobile phones have become important devices for the population, facilitating access to financial services or providing health and agricultural assistance (Pew Research Center, 2018). In these countries, market research firms generally use mobile methodologies for data collection (Robbins, 2011; Drew and Berney, 2015). This is also true for millennial consumers (born between 1981 and 1999), who generally use new technologies, especially mobile phones, more intensively (IAB, 2017). The perception of benefits that market research executives have from using consumer data collection techniques on mobile devices compared to other techniques will positively determine their use for market research by organizations. Based on the above considerations, we propose the following hypothesis:

H1: The perception of the benefits of mobile methodologies in market research by market research companies’ managers positively affects a firm’s intention to adopt mobile market research methods.

However, some characteristics of technological innovations do not always have a positive influence on adoption. The importance of business concerns related to technological innovations has been shown in previous studies (Chau and Tam, 1997; Hsu et al., 2014). Business concerns refer to perceived limitations, risks or problems that a firm can encounter when adopting innovations. The complexity of certain technological innovations, such as cloud computing, data warehouses, application service providers (ASP) and even, initially, electronic data interchange (EDI) have hindered adoption by firms (Rogers, 1995). Chau and Tam (1997), in their study on the adoption of open systems, note that perceived technological barriers in innovation determine (in this case negatively) their implementation in firms.

The literature on mobile research also highlights concerns related to this technique today. In the case of mobile surveys, one consistent finding is that response rates are lower, breakoff rates higher and completion times longer than for surveys completed on computers. Higher breakoff rates and longer completion times for mobile surveys may be due to the context of use of mobile devices (Couper et al., 2017). They can be used in a wide variety of different settings: with or without physical mobility, in different locations, in the presence of other people, and in multitasking behavior, etc. This increases the level and types of possible distractions. The low response rates observed mainly in the early years may have been due to a lack of mobile-optimized designs (Callegaro et al., 2015; Couper and Peterson, 2015; Mavletova and Couper, 2015). Callegaro et al. (2015) estimate that breakoff rates in mobile studies are two to three times higher than in questionnaires answered on desktops or laptops. Other researchers suggest that long mobile questionnaires (over 10 min) increase the likelihood of breakoff (De Bruijne and Wijnant, 2013; Mavletova, 2013), thereby providing lower response rates and poorer quality data (Buskirk and Andres, 2012; Mavletova and Couper, 2014; Wells et al., 2014). Thus, short and simple questionnaires are required (Turbina, 2014; Van Heerden et al., 2014; Mavletova and Couper, 2015). Regarding the disadvantages of mobile data collection, Poynter et al. (2014) argue that issues of security, ethics and privacy can become areas of concern in research with mobile devices. Other studies presented by Callegaro et al. (2015) and Mavletova and Couper (2015) suggest that research conducted solely on mobile devices can cause problems of representation in the sample, especially when the study population lacks the technology to take part or does not have the necessary skills (such as among the elderly). Regarding the extent to which the perception of these limitations to mobile methodologies can become barriers to adopting them over other techniques, we propose the following hypothesis:

H2: The perception of the limitations of mobile methodologies in market research by market research companies’ managers negatively affects a firm’s intention to adopt mobile market research methods.

### Organizational Context

Numerous studies on the adoption of technological innovations show that, for firms, having workers with professional competence, knowledge or expertise in innovation is positively associated with adoption. For example, Thong (1999), along with Premkumar and Roberts (1999), and Lin and Lee (2005), conclude that organizations with employees who specialize in the innovations they wish to implement are more likely to adopt them. In their study on the adoption of open systems, Chau and Tam (1997) confirm that lack of professional competence is an inhibitory factor to implementing and developing a technological innovation in an organization. Firms may even decide to put off adopting the innovation until they have sufficient internal professional competence (Thong, 1999). The market research sector has found evidence that lack of expertise or knowledge in conducting mobile market research is a possible reason, indicating why these methods have not progressed as much as forecast. Indeed, a number of authors argue that there has been a general lack of knowledge regarding how far mobile technology has developed for use in market survey data collection or that there has been insufficient professional competence to develop research with these new methods (GreenBook, 2014, 2015; Macer, 2014). Consequently, this factor is considered to have a significant impact on the adoption of mobile market research. Hence, the following hypothesis is proposed:

H3: Having company managers with professional competence in the use of mobile methodologies in market research positively affects a firm’s intention to adopt mobile market research methods.

Tornatzky and Fleischer (1990), among other researchers who defend integrated models, highlight factors and processes related to organizations and organizational structure as determinants for adopting innovation. Among these factors, a business culture with an open attitude to change, which includes considering new ideas, is an important variable in determining adoption of new technology. In the context of mobile technologies, Camponovo et al. (2005), Schierholz et al. (2007); Awasthi and Sangle (2013), and San-Martin et al. (2016) note a consistently positive influence of an open attitude to change on decisions to adopt these technologies. However, a number of academics and experts in the market research industry point out a characteristic resistance to methodological changes among industry professionals. Chang and Krosnick (2009) argue that “resistance to new modes of data collection is nothing new in the history of survey research, and it is as apparent today as it has been in the past” (p. 2). The authors claim that, much as the industry was reluctant to accept the telephone when it became an alternative to face-to-face interviews, many researchers also had doubts about switching to Internet-based data collection when representative population samples were required. Macer and Wilson (2017), in their study “Observations from 12 years of an annual market research technology survey,” discuss the slow adoption and diffusion of new technology-based methods by industry and argue that although many research firms claim to be open to new technologies, they tend to adopt a passive approach to technological development. A number of reports on changes in the market research industry, such as those produced by GreenBook and FocusVision, also point in this direction (GRIT Report, 2014 Q1-Q2; GRIT Report, 2014 Q3-Q4; GRIT Report, 2015 Q1-Q2; FocusVision MR Technology Report, 2015). In light of the above, the following hypothesis is proposed:

H4: Having organizational openness positively affects a firm’s intention to adopt mobile market research methods.

The level of satisfaction with existing systems also plays a significant role as far as motivation to change is concerned (Chau and Tam, 1997). Evidence in the literature suggests firms with a low level of satisfaction with existing systems are more open to finding new ways to improve performance or effectiveness (Rogers, 1983). This is shown in the studies by Chau and Tam (1997), Ebrahim et al. (2004), and Shih et al. (2008). Based on data published by institutions such as Esomar and GreenBook on the level of use of mobile research in the industry, it is clear that online research has maintained its predominant position over the years as the method for conducting market research. Despite the potential advantages of mobile devices, some experts maintain that there are still certain aspects in which online research is more useful, such as providing a broader variety of question formats, higher response rates, shorter response times and lower breakoff rates (GreenBook, 2015; Wells, 2015). Some sector reports support this variable as a potential disincentive to adopting mobile technologies and quote the opinions of professionals who state that while online research methods generate more confidence, it would not be difficult for mobile research to obtain universal acceptance (GRIT Report Q3-Q4, 2014; GRIT Report Q1-Q2, 2015). Based on this, we propose the following hypothesis:

H5: Having satisfied company managers with traditional and online methodologies in market research negatively affects a firm’s intention to adopt mobile market research methods.

Larger firms are usually more likely to adopt innovation if they have more resources and infrastructures to do so (Utterback, 1974; Moch and Morse, 1977; Dewar and Dutton, 1986). Business size is considered a positive determinant in adopting innovation in the case of email and the Internet (Premkumar and Roberts, 1999), e-business (Zhu et al., 2003, 2006; Bordonaba-Juste et al., 2012), electronic signature (Chang et al., 2007), enterprise resource planning (ERP) (Pan and Jang, 2008), cloud computing (Low et al., 2011), and e-collaboration technology (Chan et al., 2012). However, it is also considered a significant negative influence in adopting certain technological innovations, such as e-business by financial companies (Zhu et al., 2004), wireless identification technologies in the health industry (Chong and Chan, 2012), and ERP systems (Junior et al., 2019). So, although size is widely studied, a conclusive link between this factor and innovation does not exist (Baker, 2011). The literature on market research makes no special mention of size as a precursor to the use of new market research methods or technologies. However, professionals in the sector claim that new technological developments are created by both small – though highly dynamic and innovation-oriented – firms, and in the majority of large firms, as well as at some specialist technology developers (Macer and Wilson, 2017). Robbins (2011) and Poynter et al. (2014) argue that the use of mobile research techniques can involve costs associated with developing, programming and testing questionnaires adapted to mobile devices, training organization personnel and contracting experts. Thus, larger or multinational firms are more likely to adopt innovative methods. Given that the size factor is included in much of the research into technology adoption by organizations, we consider it appropriate to include it here as well. However, given the insufficient evidence in the literature on mobile research, and following Hair et al. (2015), who state that non-directional hypotheses must be considered if there are limited or ambiguous findings in the literature regarding the effect of the independent variable on the dependent variable, we propose the following hypothesis that does not establish a direct relationship (positive or negative) between variables:

H6: Firm size has an influence on the intention to adopt mobile market research.

### Environmental Context

The environmental context is defined as the scenario in which an organization conducts its business, including the members of the industry, regulatory authorities, clients and suppliers (Tornatzky and Fleischer, 1990; Zhu et al., 2003). These factors may stimulate adoption and diffusion of innovation within an organization as a response to competitive pressure, regulatory actions and customer satisfaction requirements. Thus, organizations can adopt a given technology voluntarily or due to the influence of other firms in the industry (Srinivasan et al., 2002) or from clients (Wu et al., 2003), who may demand the use of such technology when contracting services. Prior research on communications technology has shown that it has become a strategic necessity to have these technologies in order to compete in the marketplace (Premkumar and Roberts, 1999). Many firms adopted information systems due to demand from clients to improve the efficiency of their interorganizational transactions (Low et al., 2011; Gutierrez et al., 2015). In the field of mobile market research, Robbins (2011) stresses that the incorporation of mobile methodologies in a firm’s service portfolio can provide a differentiating factor, making it more competitive. This implies that adopting such methods can provide strategic, differential added value in the industry, as firms may be perceived as innovative by their competitors and clients. Muñinos (2019) also refers to possible pressure from competitors to adopt technological innovations in the market research industry and notes that the more firms that implement innovation in certain processes, the greater the number of firms that will follow (p. 16). With regard to whether the clients who contract market research are an influence on the choice of information collection methods, the industry claims that a large number of clients choose to design short questionnaires adapted to mobile screens for mobile surveys (GRIT Report Q3-Q4, 2015). Based on the above, the following hypotheses are proposed:

H7: The perception of pressure from the market research industry by market research company managers positively affects a firm’s intention to adopt mobile market research methods.

H8: The perception of pressure from clients by market research company managers positively affects a firm’s intention to adopt mobile market research methods.

With the dramatic rise in mobile devices’ diffusion worldwide since 2010, the trend in mobile research has come about ‘by accident,’ ‘unintentionally’ or ‘involuntarily’ (Peterson et al., 2013). Thus, research subjects were answering surveys they received via their panel platform or by email on their mobile phones, even though they were not optimized for such devices. Researchers saw this phenomenon, which occurred above all between 2010 and 2014, as an emerging concern. The percentage of surveys with non-responsive designs completed on mobile devices increased every year and in hundreds of market research studies carried out in the USA, Europe and South America (Revilla et al., 2015), reaching rates of over 40% in some cases (Wells, 2015). The trend showed that the boom in mobile technologies and their intensive use were significant factors, which meant that the industry needed to pay more attention to mobile methodologies. The fact that survey participants are increasingly using their mobile terminals to respond has been considered influential in the adoption of mobile methodologies in market research (Ochoa and Castro, 2015; Macer and Wilson, 2017). Studies also suggest that pressure from survey participants has forced firms to accelerate their adoption of mobile market research (op cit). Since pressure from participants in market research surveys can be considered - specifically for the subject of this research – as a factor of external pressure included in the environmental context of the TOE framework, this study hypothesizes the following:

H9: The perception of pressure from survey participants by market research company managers positively affects a firm’s intention to adopt mobile market research methods.

**FIGURE 1:**
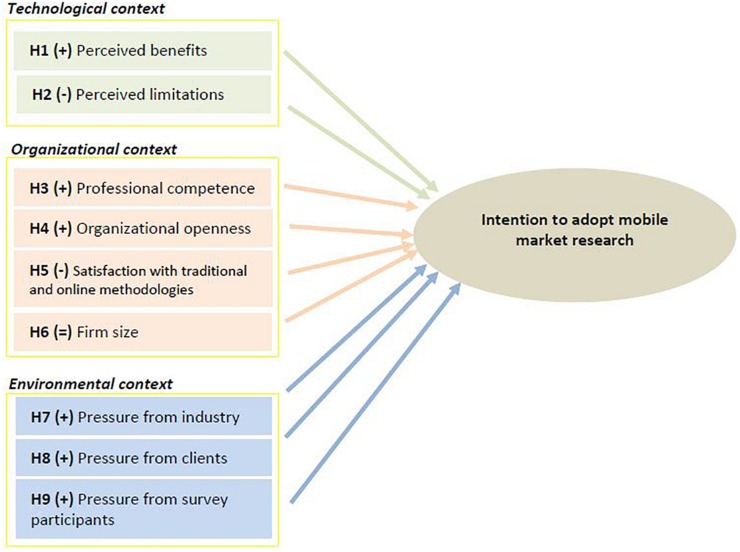
Research model and hypothesis.

The research model is shown in Figure 1. The proposed model builds on the TOE framework, outlining the role of technological, organizational and environmental variables as key factors influencing market research companies’ intention to adopt mobile market research in their projects.

## Research Method

A qualitative study was initially conducted due to the lack of literature on mobile market research adoption in relation to some of the constructs included in the model. First, we carried out an exhaustive analysis of the contents of the main sector-based reports published by market research institutions (such as Esomar, GreenBook, Confirmit, Aedemo, and Aneimo). The information in these reports was used to develop a theoretical and conceptual research framework and some of the hypotheses proposed in the theoretical model. Secondly, seven in-depth interviews were conducted with experts in online and mobile market research, all of whom work for the main market research firms in Spain and have over 20 years’ experience in the industry. The purpose of this qualitative phase was to provide a deeper understanding of the problem being studied, refine the structure of the items proposed to measure some of the constructs of the model, and improve the definition of the survey questions. It also helped define the research sample design and execute the fieldwork in the subsequent quantitative phase, as it provided information on the size of the market research sector in Spain and contacts to help distribute the questionnaire used in the quantitative study.

The survey instrument used in the quantitative study was constructed from established construct measures in the literature on adoption of technological innovations and mobile market research, adapted to ensure its applicability to the context of our proposed model. New measurement items from the qualitative phase were included to measure a number of the constructs, these being: perceived limitations, satisfaction with traditional and online methodologies, and constructs related to environmental pressure (*pressure from the industry*, *clients* and *survey participants*). With the exception of the *firm size* construct, Likert scales were developed to measure the latent variables of the model. All items were anchored on a 7-point Likert scale (1 = strongly disagree; 7 = strongly agree). Table 1, contains the complete details of the full set of measurement scales used for each construct.

An online survey was carried out to collect the data. After initial testing, the final questionnaire, with a responsive design for completion on mobile devices, was sent to a population of 180 Spanish market research firms from the main Spanish market research association (Aedemo). Eligible respondents were mid-to-senior level decision-making market research professionals, as they are responsible for choosing the research methodology used in each project. Convenience and snowball sampling were used to collect a final total of 67 complete sets of answers, representing a response rate of 37.2%.

## Data Analysis and Results

The data from the study were analyzed using partial least squares (PLS), a structural equation modeling technique. This is a powerful technique for analyzing topics that have not been tested before (Teo et al., 2008; Hair et al., 2011), when small samples are used, as it does not make relative assumptions regarding data distribution (Chin and Newsted, 1999; Chin et al., 2003; Hair et al., 2017). In our case, we obtained a sample of 67 interviews. PLS was deemed appropriate given that this study is an initial attempt to explain the intention to use mobile market research, and to identify the key determinants influencing this adoption. Hair et al. (2011) recommend using PLS-SEM “if the goal is predicting key target constructs or identifying key ‘driver’ constructs” (p. 144), as in our case. Similarly, other authors suggest that PLS-SEM is appropriate when the research has a predictive purpose (Cepeda et al., 2016; Shmueli et al., 2016) and an explanatory purpose (Henseler, 2018), as is the case with our study. Smart PLS 3.0 (Ringle et al., 2015) was used to evaluate the reliability and validity of the measurement model and analyze the structural model. Table 2, shows the means and standard deviations of the model constructs.

**TABLE 2:** Descriptive statistics.

	**Construct**	**Code**	**Number of indicators**	**Mean**	**Standard deviation**
Technological factors	Perceived benefits	PB	6	5.80	1.369
	Perceived limitations	PL	4	5.07	1.618
Organizational factors	Professional competence	PC	4	4.35	1.751
	Organizational openness	OO	4	5.51	1.341
	Satisfaction with traditional and online methodologies	SAT	3	4.84	1.327
	Firm size	DIM	3	n/a	n/a
Environmental factors	Pressure from industry	IP	6	5.49	1.311
	Pressure from clients	CP	4	4.66	1.476
	Pressure from participants	PP	4	5.53	1.333
Dependent variable	Intention to adopt mobile market research	ADO	2	5.70	1.359

### Sample Characteristics

The profile of the sample respondents is given in Table 3, including firm size, scope, proprietary panel and mobile research usage. Firms with fewer than 10 employees represented 58.2% of the sample, while the remaining 41.8% had 10 or more. A total of 10.9% of the firms belonged to an international group and 35.8% had a proprietary panel. Regarding the use of mobile research techniques, most of the firms (85.1%) stated that they used online questionnaires (adapted to mobiles). Mobile CAPI and mobile CATI were used by 63.2 and 57.5% respectively. 34.3% of the firms in the sample used passive data in projects - the same percentage as firms using mobile-only questionnaires. With regards to qualitative techniques, 51.2% of firms stated that they used mobile ethnography, while 48.4% stated that they used mobile devices for other qualitative methods (such as in-depth interviews, focus groups and research communities). In terms of the number of methods used, half the firms stated that they used between two and four mobile techniques. On average, the number of mobile methodologies used by market research firms was 3.7.

**TABLE 3:** Profile of sample respondents.

**Profile category**		**Percentage %**
Size	Fewer than 10 employees	58.2
	10 or more employees	41.8
Scope	Spanish firm	89.1
	International group	10.9
Proprietary panel	No proprietary panel	64.2
	Proprietary panel	35.8
Mobile methodology usage	Online surveys	85.1
	mCAPI	63.2
	mCATI	57.5
	Mobile ethnography	51.2
	Mobile qualitative research	48.4
	Passive data	34.3
	Mobile-only surveys	34.3

### Results

#### Measurement Model

Before testing the structural relationships of the theoretical model, the measurement model was verified to ensure it provided the necessary conditions of reliability and convergent and discriminant validity. Table 4, shows the results of the measurement model. The three indicators used to validate the reliability of the measurement instrument were the Cronbach α and KR20 coefficients (Cronbach, 1951; Nunnally and Bernstein, 1994; critical acceptance value = 0.7), composite reliability index (Fornell and Larcker, 1981; critical acceptance value = 0.7, or Bagozzi and Yi, 1988; critical acceptance value = 0.6) and average variance extracted (AVE) (Fornell and Larcker, 1981; critical acceptance value = 0.5). These three reliability indicators exceed the corresponding critical values for each of the factors, except Cronbach’s α for the construct ‘satisfaction with traditional and online methodologies’, which has a value below 0.6. However, the composite reliability value for this construct is 0.70 and, furthermore, the AVE is greater than 0.50, thus it was decided to be kept in the model. As evidence of convergent validity, the results indicate all items are significantly (*p* < 0.01) related to their hypothesized factors and the size of all standardized loadings are higher than 0.60 (Bagozzi and Yi, 1988). Except for two measures of constructs, all items loaded on their hypothesized variables. In the case of ‘professional competence’ and ‘organizational openness’, the items loaded on the same factor. Moore and Benbasat (1991); Benham and Raymond (1996), and Thong (1999) had a similar experience in the empirical testing of their studies. Although there may be a conceptual difference between these two constructs, they are perceived identically by respondents. In this study, we combined the items measuring these two closely related organizational variables into a single variable. For the subsequent statistical analysis, the score for each composite research variable was the aggregate of a respondent’s scores for items defined to measure that variable. In testing the measurement model, evidence for discriminant validity of the measures (see Tables 5, 6) was tested checking that the shared variance between pairs of constructs was less than the corresponding AVE (Fornell and Larcker, 1981). Based on these criteria, we conclude that the measures in the study provide sufficient evidence of reliability and convergent and discriminant validity.

**TABLE 4:** Results of the measurement model.

**Factor**	**Item**	**Convergent validity**	**Reliability**		
		**Loadings**	**Cronbach α**	**CR**	**AVE**
Perceived benefits(PB)	PB1	0.731	0.902	0.923	0.667
	PB2	0.846			
	PB3	0.764			
	PB4	0.798			
	PB5	0.829			
	PB6	0.920			
Perceived limitations (PL)	PL1	0.746	0.839	0.884	0.657
	PL2	0.763			
	PL3	0.840			
	PL4	0.885			
Professional competence/organizational openness (PCOO)	PC1	0.646	0.922	0.925	0.611
	PC2	0.738			
	PC3	0.645			
	PC4	0.616			
	OO1	0.933			
	OO2	0.844			
	OO3	0.867			
	OO4	0.889			
Satisfaction with traditional and online methodologies(SAT)	SAT1	0.726	0.511	0.753	0.504
	SAT2	0.720			
	SAT3	0.682			
Firm size(DIM)	DIM1	0.657	0.779^(1)^	0.866	0.687
	DIM2	0.851			
	DIM3	0.952			
Pressure from industry(IP)	IP1	0.659	0.851	0.889	0.575
	IP2	0.740			
	IP3	0.867			
	IP4	0.887			
	IP5	0.793			
	IP6	0.804			
Pressure from clients(CP)	CP1	0.785	0.739	0.835	0.560
	CP2	0.799			
	CP3	0.645			
	CP4	0.755			
Pressure from participants(PP)	PP1	0.859	0.826	0.885	0.658
	PP2	0.789			
	PP3	0.798			
	PP4	0.796			
Intention to adopt(ADO)	ADO1	0.868	0.701	0.870	0.769
	ADO2	0.887			

*Convergent validity and reliability. CR, composite reliability; AVE, average variance extracted. (1) Kuder and Richardson 20 (KR20) formula has been used for firm size reliability measurement.*

**TABLE 5:** Results of the measurement model.

	**ADO**	**PC/OO**	**PB**	**DIM**	**PL**	**CP**	**IP**	**PP**	**SAT**
ADO	0.877								
PC/OO	0.346	0.781							
PB	0.276	0.057	0.817						
DIM	0.184	0.208	0.140	0.829					
PL	0.175	−0.295	0.036	0.085	0.811				
CP	0.684	0.177	0.272	0.254	0.206	0.749			
IP	0.689	0.073	0.340	0.249	0.367	0.718	0.758		
PP	0.669	0.040	0.349	0.274	0.162	0.674	0.725	0.811	
SAT	−0.440	−0.190	−0.102	−0.231	−0.015	0.399	−0.250	−0.334	0.710

*Discriminant validity. Diagonal of the matrix: square root of the AVE for each construct. Below the diagonal: bivariate correlations between constructs.*

**TABLE 6:** Results of the measurement model.

	**ADO**	**PC/OO**	**PB**	**DIM**	**PL**	**CP**	**IP**	**PP**	**SAT**
ADO									
PC/OO	0.320								
PB	0.310	0.176							
DIM	0.214	0.259	0.179						
PL	0.197	0.425	0.146	0.170					
CP	0.924	0.174	0.316	0.334	0.236				
IP	0.869	0.211	0.410	0.316	0.430	0.865			
PP	0.875	0.087	0.402	0.333	0.226	0.848	0.858		
SAT	0.724	0.293	0.201	0.331	0.219	0.631	0.368	0.541	

*Heterotrait-monotrait (HT/MT) ratio.*

#### Structural Model

The estimation of the parameters was obtained using a bootstrapping procedure of 5,000 samples to calculate the significance of the path coefficients. Table 7, presents the results of the hypotheses testing. The proposed model explained 68.4% of the variance in intention to adopt mobile methodologies in market research. Based on the analysis, five of the nine hypotheses in the model were supported. Among the technological constructs, the model estimation showed there was no significant relationship between perceived benefits and perceived limitations and the intention to adopt mobile market research (H1 and H2 were not supported). Regarding organizational context, professional competence in mobile methodologies and an open attitude in the firm toward change exert a significant positive effect on intention to adopt (β = 0.300, *p* < 05; H3 and H4 supported). As for the influence of satisfaction with traditional and online collection methods on intention to adopt, a negative effect is confirmed (β = −0.171, *p* < 05; H5 supported). However, firm size had no significant relationship with intention (H6 was not supported). Finally, with regard to the environmental constructs, pressure from industry (β = 0.282, *p* < 05; H7 supported), clients (β = 0.172, *p* < 10; H8 supported) and participants in the surveys (β = 0.295, *p* < 05; H9 supported) had a significant positive influence on intention to adopt mobile market research. We use PLSPredict with two folds and 10 repetitions to obtain the model out-of-sample predictive power (Cepeda et al., 2016; Shmueli et al., 2016). We report the prediction statistics of the endogenous constructs’ indicators in Table 8. In a first step, we find that the endogenous constructs’ indicators yield Q^2^_predict_ values above 0. Next, we analyze the prediction errors in detail to identify the relevant prediction statistic, which suggest that the distribution is not highly non-symmetric. Hence, we base our predictive power assessment on the RMSE. Comparing the RMSE values from the PLS-SEM analysis with the LM benchmark, we find that the PLS-SEM analysis produces lower prediction errors for the indicators. According to Shmueli et al. (2019), these results confirm a high predictive power of the model.

**TABLE 7:** Results of hypothesis testing.

**Hypothesis**	**Structural relationship**	**β**	***t* Bootstrap**	**Contrast**
H1	Perceived benefits → Intention to adopt	0.009	0.061	Not supported
H2	Perceived limitations → Intention to adopt	0.084	0.832	Not supported
H3 + H4	Professional competence + organizational openness → Intention to adopt	0.300	1.930**	Supported
H5	Satisfaction with traditional and online methodologies → Intention to adopt	−0.171	2.159**	Supported
H6	Firm size → Intention to adopt	−0.121	1.413	Not supported^1^
H7	Pressure from industry → Intention to adopt	0.282	2.212**	Supported
H8	Pressure from clients → Intention to adopt	0.172	1.344*	Supported
H9	Pressure from participants → Intention to adopt	0.295	2.172**	Supported

* *p* < 0.1; ** *p* < 0.05. *R*^2^ (Intention to adopt) = 0.684. ^1^As firm size is part of a hypothesis with no positive or negative direction, a two-tailed significance test was conducted. Thus, with a *t* value = 1.413 and 4999 degrees of freedom, the *p* value is 0.157718. The result is not significant at the accepted significance levels (0.01, 0.05, 0.10).

**TABLE 8:** PLSPredict assessment of the endogenous variable.

**Item**	**PLS SEM**		**LM**	**PLS SEM -**
	**RMSE**	**Q^2^_predict_**	**RMSE**	**LM RSME**
ado1	1.225	0.336	1.320	−0.095
ado2	0.971	0.442	1.049	−0.078

## Discussion and Conclusion

### Discussion

The study identified professional competence and a firm’s openness to change, pressure from participants in market research, from the industry and from clients, and satisfaction with traditional and online methodologies as important variables for affecting mobile market research adoption. Nevertheless, the results confirm that the perceived benefits of mobile data collection, which represent advantages over other market research techniques, do not influence its use. Despite abundant empirical evidence of the weight of perceived benefits in adopting numerous technological innovations (EDI, Internet, email, e-commerce, e-business, ERP, cloud computing, SaaS), our research clearly shows that the benefits provided by technological features inherent in the innovation (speed in data collection, convenience for participants, less memory bias, access to difficult-to-access populations, passive data collection, new approaches in ethnographic studies) are not a driver in its use. This conclusion was also reached by a number of studies that analyzed adoption of e-business (such as Zhu et al., 2003; Xu et al., 2004; Bordonaba-Juste et al., 2012), electronic signature (Chang et al., 2007), e-collaboration technologies (Chan et al., 2012), and mobile commerce (San-Martín et al., 2012). Nor did the findings support a negative influence of barriers associated with mobile market research in its adoption. The result shows that although professionals are aware of possible problems in using mobile market research methods in studies (especially those related to the length and design of certain questions on questionnaires and the greater risk of breakoff), these are not empirically determinant factors in hindering the use of mobile research. This result differs from other studies on the adoption of technological innovations, such as cloud computing systems and data warehouses, where existing technological barriers to implementing such systems were considered a significant determining factor with a negative influence on adopting such innovations. This is an interesting finding and contradicts previous research, as technological factors associated with mobile technology itself are no longer a fundamental and decisive factor in the specific field of adopting mobile market research. This suggests that mobile technology is not a disruptive innovation in the sector (unlike the effect of the preceding technology, online research, on traditional information collection) and that other factors determine its adoption.

The level of professional competence in the firm and openness to change, which are linked to a single construct, jointly reflect the positive effect of both in adopting mobile research. Indeed, variables in the ‘professional competence’ and ‘organizational openness’ constructs could not be differentiated in the analysis. This may indicate that, if a firm ensures it has staff able to meet the business challenges it faces and an organizational culture that promotes an open attitude to change, both factors may be mutually influential. Furthermore, the research shows that the degree of professional competence and organizational openness largely determines the use of mobile techniques in market research. Thus, firms with an open attitude to introducing change in their practices by creating and/or adopting new ideas and which also have professionals who are competent in the most innovative data collection methods are those that generally decide to carry out research using mobile devices to collect data on the behavior of the target population. This conclusion further suggests that attitudes which are less open or even resistant to change (as characterized by the market research industry worldwide and in Spain) lead to lower levels of adoption of technological innovations. In fact, both professional competence and an attitude of openness to change are reported in a number of studies as important determinants in the adoption of technological innovations (Chau and Tam, 1997; Thong, 1999; Lin and Lee, 2005).

A higher level of satisfaction with traditional and online data collection techniques was found to have a negative effect on adopting mobile market research methods. High levels of satisfaction lead to a perception that mobile research is not particularly necessary. This finding is in line with the research of previous studies, which found satisfaction with previous systems was a negatively correlating determinant in adopting innovations (Chau and Tam, 1997; Ebrahim et al., 2004; Shih et al., 2008). Data published by Esomar and GreenBook show that online research has remained a predominant method in recent years in the industry, representing 30% of expenditure in 2017 (ESOMAR European Society for Opinion and Marketing Research, 2018) and used in almost 60% of studies (GreenBook, 2018). Despite a downward trend, face-to-face and telephone surveys and a number of qualitative techniques (such as face-to-face group discussions and online research communities) still represent a considerable percentage of expenditure and use. Online research is therefore still the most trusted method in the market research industry to meet the clients’ research goals, given its proven effectiveness in collecting data from participants.

With regard to firm size, as no evidence was found to indicate this factor is a positive or negative element contributing to the adoption of mobile market research methods, the hypothesis was not considered to have established a directional relationship, but it does have an influence on the endogenous variable. However, the results show the construct does not support the proposed hypothesis, i.e., a significant relationship is not found between both variables. Our research therefore shows that the smallest firms in the industry are just as likely to adopt mobile research as larger firms. In addition, structural inertia in the market research industry could cancel out firm size as a determinant in adopting mobile research. This has also been found in other studies on technological innovation adoption, such as Zhu et al. (2004) and Tan et al. (2007).

This study proposes the hypothesis that three environmental elements have a direct and positive influence on firms in the industry and lead them to adopt mobile research methods. The three environmental elements are: (1) pressure from industry; (2) pressure from clients contracting market research; and (3) pressure from survey participants. The findings indicate that the perception of pressure from industry positively influences the use of research techniques with mobile devices. This result corroborates previous studies (such as Iacovou et al., 1995; Teo et al., 1997; San-Martin et al., 2016), in which pressure from competitors was identified as a driver for adoption. In this context, adopting technological innovations is an unavoidable element for a firm striving to be competitive, and is thus a necessity for firms in the market research industry. This research shows that competitive pressure means that there is a greater likelihood to adopt research methods with mobile devices.

Perceived pressure from clients to whom firms offer their services is also a factor affecting mobile research (although the significance level is 10%), exercising a positive influence on the use of mobile research techniques. This other element in the business environment is also a determining factor in adopting a variety of other innovations, such as e-business (Srinivasan et al., 2002; Wu et al., 2003), B2C e-commerce (Ching and Ellis, 2004; Lee and Kim, 2007; Rodríguez-Ardura et al., 2008) and cloud computing systems (Low et al., 2011; Gangwar et al., 2015). Finally, a specific factor included in this study, the pressure exerted by participants in market research, is the second most relevant factor in adopting mobile research. The popularity of smartphones among the population and their increasing use for all activities of daily life was an unexpected phenomenon in mobile research. As it occurred, the industry hesitated over the best strategy to adapt to this new reality. While some firms did not take long to adopt the ‘mobile first’ design in their studies, others went so far as to prohibit participants from answering their questionnaires on mobile devices. Almost all firms and professionals in the industry are now aware of the need for questionnaires optimized for mobile screens, thus confirming the importance of this element as a driver for the use of mobile methodologies in market research. Indeed, as Ochoa and Castro (2015) affirm, mobile research should be at the disposal of the research subject and not the researcher. In this respect, businesses in the market research sector should use mobile methods in their projects, as they are more convenient for participants who find it easier, more comfortable and faster to take part in research via their mobile devices. Mobile technologies are starting to exercise a pull effect upon market research firms, who see the need to use data collection techniques that are well adapted to increasingly technologically oriented client profiles and to ensure their research process is engaging and valuable for participants.

### Conclusion

We can conclude that the findings are in line with previous research studies, thus underlining the usefulness of the TOE model. However, and contrary to that expected, this study shows that the technological factors most frequently used in academic research involving TOE theory and which have been empirically confirmed as determinants in technological innovations do not, in our case, represent drivers or barriers for adopting mobile methodologies in market research. In other words, neither the perceived benefits nor perceived limitations associated with mobile technology are predictors for the intention to use or not use mobile research. This means that other organizational and environmental factors are the main influences on the intention to use mobile technology in the industry.

The market research industry should bear in mind that it might well be a strategic error to ignore or respond only nominally to the opportunities that different technological revolutions can represent: technology provides and exponentially augments data collection capacity and as such, the industry faces a new challenge – the need to analyze this wealth of data. In fact, our research demonstrates that the most influential factor in the adoption of market research via mobile devices is openness to change and possessing the necessary talent to facilitate the management of different technological innovations. In terms to this latter factor – relative to professional competence – those firms that do not have their own technological development teams should aim to bring in personnel specializing in this area or, when they lack the resources to do so, at least increase their involvement in working with external technology providers. According to the results obtained in this study, this could help advance toward the adoption of mobile-based research methods.

In addition, pressure exerted by the environment can be confirmed as relevant in the intention to adopt mobile market research. More specifically, pressure exerted by research participants is identified as one of the most important determinants in adopting mobile technology. On this basis, the industry should no longer consider mobile methodologies in market research as a separate research technique. They should be viewed as a new medium that allows greater alignment with participants and, in turn, makes participants feel more involved and invested as collaborators in data collection and generation of insights. Commitment to adopting such methods on a strategic level can generate differential added value for firms in the market research industry in terms of being perceived and understood as innovative both by their competitors and by their clients.

## Data Availability Statement

The datasets for this manuscript are not publicly available because commercial confidentiality. Requests to access the datasets should be directed to CP-B, mpachecob@uoc.edu.

## Ethics Statement

This study was approved by the Universitat Oberta de Catalunya (UOC) Research Ethics Board and according to ICC/ESOMAR International Code on Social Research. Each participant provided written informed consent.

## Author Contributions

CP-B developed the theoretical framework, performed the analysis, and wrote the manuscript with support from AJ-Z and M-JM-A. All authors discussed the results, provided critical feedback, and contributed to the final version of the manuscript.

## Conflict of Interest

The authors declare that the research was conducted in the absence of any commercial or financial relationships that could be construed as a potential conflict of interest.
